# Experimental evaluation and constitutive modelling of cervical spine ligaments under low and high strain rates

**DOI:** 10.1038/s41598-025-23918-8

**Published:** 2025-11-17

**Authors:** Radosław Wolny, Tomasz Wiczenbach, Lukasz Pachocki, Angela Andrzejewska-Sroka, Agnieszka Sabik, Błażej Meronk, Karol Daszkiewicz, Magdalena Rucka, Wojciech Witkowski, Edyta Spodnik, Jan Henryk Spodnik, Ilya Krypets, Piotr Łuczkiewicz, Krzysztof Wilde

**Affiliations:** 1 Department of Mechanics of Materials and Structures, Faculty of Civil and Environmental Engineering, Gdańsk University of Technology, Narutowicza 11/12, Gdańsk, 80-233 Poland; 2 Division of Anatomy and Neurobiology, Department of Anatomy, Medical University of Gdańsk, Gdańsk, 80-210 Poland; 3 II Clinic of Orthopaedics and Kinetic Organ Traumatology, Medical University of Gdansk, Smoluchowskiego, Gdańsk, 80-210 Poland

**Keywords:** Human cervical spine ligaments, Strain‑rate dependent mechanical properties, Visco-hyperelastic constitutive law, Biomechanics, Soft materials, Theory and computation, Biomedical engineering

## Abstract

In the paper the mechanical behavior under low (0.5 s⁻¹) and high-strain rate (50 s⁻¹) of four human cervical spine ligaments, anterior longitudinal ligament (ALL), posterior longitudinal ligament (PLL), ligamentum flava (LF), and capsular ligament (CL), harvested from elderly donors (70–90 years) is investigated. Results highlight that the PLL, LF, and CL exhibit statistically significant increases in stiffness and elastic modulus under high-strain loading. An increase in the failure force was observed only for the PLL and LF at 50 s⁻¹. Meta-regression analyses revealed that age exerts a negative influence on certain mechanical parameters, emphasizing the need for age-matched biomechanical data in both clinical and computational studies. A visco-hyperelastic constitutive model was fitted to the experimental stress-stretch curves, capturing both low-strain and high-strain rate responses with high goodness-of-fit metrics. Presented results advance understanding of ligament mechanics in older populations and offer valuable data for improving mathematical models aimed at injury prediction, particularly for high-impact events.

## Introduction

 Cervical spine ligaments, including the anterior longitudinal ligament (ALL), the posterior longitudinal ligament (PLL), the ligamentum flava (LF), and the capsular ligament (CL), are critical for maintaining the structural integrity and functionality of the neck^1^. They can be subjected to complex mechanical forces with strain-rates varying from relatively low, typical for usual daily activities, to high, occurring in sudden, high-velocity events like, e.g., sport-related impacts, falls, or car crash accidents. Sudden and unexpected spine movements that exceed the physiological range can cause ligament damage^2^. Thus, accurate identification of specific stress and strain thresholds that lead to ligament rupture is crucial for the effective design of spinal protection devices and safety features in vehicles, such as seat belts and headrests^3,4^.

To date, the mechanical response of human cervical spine ligaments has been studied under low^1,5–7^ or high-strain rate^8,9^. However, the analysis of the influence of strain rate on their properties is limited^10–12^. In^10^ it was considered for a younger population only. While in^11^ relatively low-strain rates were investigated, and in^12^ only two ligaments from an unspecified population were investigated. Consequently, despite their increased susceptibility to injury, older individuals have received comparatively less attention in the investigation of viscous properties. This emphasizes the need to extend the study of both the strain-rate effect and ageing on ligament biomechanical properties. The ageing population presents unique challenges due to age-related changes of biomechanical properties, such as reduced elasticity, altered collagen composition, and decreased cross-sectional areas, which can affect injury patterns and recovery processes^13^. Understanding how the mechanical behavior of cervical spine ligaments changes with age is crucial for developing more effective injury prevention and treatment strategies for the elderly population, who are at a higher risk of sustaining cervical spine injuries due to factors like decreased muscle strength and balance.

Nowadays, the development of these strategies can be successfully supported by numerical simulations. Indispensable tools in this field are the human body models (HBMs), such as the Total Human Model for Safety (THUMS) and the VIVA+^4,14–16^. They aim to simulate the response of the human body under various loading conditions and are often used in analyses of advanced safety systems, like airbags or road safety barriers, etc^6^. These models provide information about the complex mechanisms underlying injury without the ethical and practical constraints associated with cadaveric testing^17^. However, the accuracy of HBMs is limited by the biomechanical data they incorporate. These limitations are related especially to the geometry and material parameters of the tissues and organs. Most existing HBMs are based on data derived from younger cadavers and simplifying assumptions, which do not adequately capture the nuanced biomechanical properties of tissues in older populations^18^. Incorporating accurate, age-specific biomechanical data into HBMs is, therefore, crucial for enhancing their predictive accuracy and ensuring their relevance across diverse anthropometric groups. Thus, age-related alterations in ligament structure and function warrant further investigation. To the best of the authors’ knowledge, previous studies have not comprehensively described the strain rate effect on the mechanics of cervical ligaments and their properties under high-strain rates typical of real-world injuries^5,6,19^ in the elderly population.

Taking the above into account, this work presents an investigation of the low-strain and high-strain rate tensile response characteristics of cervical spine ligaments from older adult cadavers^20^. The study involves experimental tensile testing of ligament samples, followed by developing a potential constitutive model that accurately matches the observed experimental data. In the authors’ opinion, the anticipated outcomes of the work can serve as a valuable support across a range of practical fields, such as e.g., the design of therapeutic and injury prevention techniques for the elderly population and improvements of vertebral column models in in silico studies, including those with HBMs^14,15,21,22^.

## Materials and methods

Gratitude to the body donors and their families is conveyed in the **Acknowledgements** in accordance with the guidelines of^23^. All cadavers analyzed in this work were acquired through a body donation program, following consent for use in educational and scientific activities. The study protocol received approval from the Institutional Ethics Committee (IRB reference: Ordinance No. 26/2016 of the Rector of the Medical University of Gdańsk, dated June 6, 2016, concerning the “Program of Conscious Donation of Corpses”). A total of seven human cervical spines (mean donor age 78 years within a range 70–90) were obtained. The donor group comprised two males and five females, with an average weight of 78 kg (range 60–90 kg) and an average height of 172 cm (range 150–190 cm). Within 24–48 h postmortem, specimens of 15 ALL, 17 PLL, 13 LF, and 29 CL were isolated. Only those ligaments without any detectable pre-existing bone or soft-tissue pathology were included. Relevant anthropometric data, specifically age, gender, weight, height, and autopsy details, were collected and recorded for each donor in Table 1.

**Table 1:** Cadaver demographic and anthropometric data

Cadaver No.	Sex, m/f	Weight*, kg	Height*, cm	Age, years	Strain rate, Low/High	Number of Samples
1	m	90	190	90	High	6
2	m	80	180	73	High	12
3	f	90	175	70	Low	11
4	f	90	175	75	Low	12
5	f	65	170	78	Low	13
6	f	70	165	84	High	6
7	f	60	150	76	High	14

*rounded to the nearest multiple of 5.

### Specimens

#### Preparation

Each spine was harvested through a posterior approach. Two sagittal skin incisions were initially made, positioned 30–40 mm lateral to the posterior midline on each side of the spine. This incision extended from the occipital bone to the sacrum. The skin flap, along with underlying subcutaneous tissue, was dissected and excised. Subsequently, the dorsal muscles were detached from their cranial attachments. The muscles spanning the spinous processes, including the dorsal extensor muscles, were systematically dissected and removed. Next, the atlantooccipital and sacroiliac joints were resected. The ribs were transected approximately 10 mm lateral to the transverse processes, and the sacrum was horizontally sectioned at the S2/S3 level. The quadratus lumborum muscles were also debrided. Following these procedures, the entire spine was extracted and examined from an anterior view, with particular attention to the diaphragm and the psoas major muscles.

The spine was then lavaged three times with chilled saline (0.9% NaCl) at approximately 6 °C, each lavage lasting 15 min. The vertebral column was segmented after removing the short muscles and excising remnants of ribs at the costovertebral joints. The isolated segment of the entire cervical spine (C2-T1, with C*i* and T*i* standing for the *i-*th vertebra of the cervical and thoracic spine, respectively) was divided into functional spine units (FSUs). First, the intervertebral discs were cut, then the ligaments were dissected, and the facet joints were excised every other level, resulting in three FSUs named as C2–C3, C4–C5, and C6–C7.

**Fig. 1:**
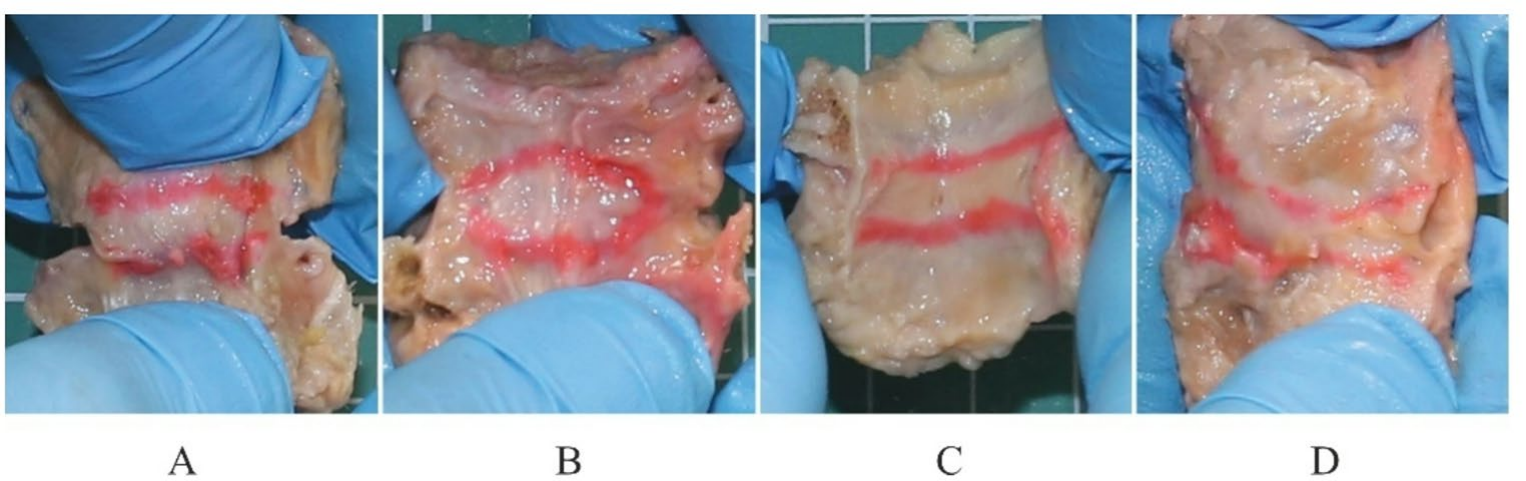
The BLB specimens. (**A**) ALL, (**B**) PLL, (**C**) LF, and (**D**) CL

Next, the obtained vertebra-disc-vertebra units were dissected to obtain the desired bone-ligament-bone (BLB) specimens (see Fig. 1). From each segment, the ALL, PLL, LF, and CL (left and right) were collected. The ligaments were then cleaned of fatty tissue. The ALL and PLL were separated from the bisected intervertebral disc. The nucleus pulposus was removed first, after which the annulus fibrosus (AF) was separated from the vertebral bodies along both the superior and inferior cartilaginous endplates (CEP). Final dissection of the AF from the deep layers of the longitudinal ligaments was initiated from the lateral aspects, taking into account morphological differences in fiber structure and orientation between the longitudinal ligaments and the AF. While the AF is characterized by a lamellar architecture with circumferentially arranged fiber bundles, the longitudinal ligaments consist of longitudinally oriented fiber bundles. The ligaments were then cleaned of fatty tissue. Preparation of the vertebral column and ligaments was performed by experienced anatomists using sharp and blunt techniques and a variety of dissection instruments. Each of the obtained samples was wrapped in multiple layers of lignin soaked in isotonic saline solution, then placed in a string bag and frozen at − 20 °C.

#### Geometry measurements

**Fig. 2:**
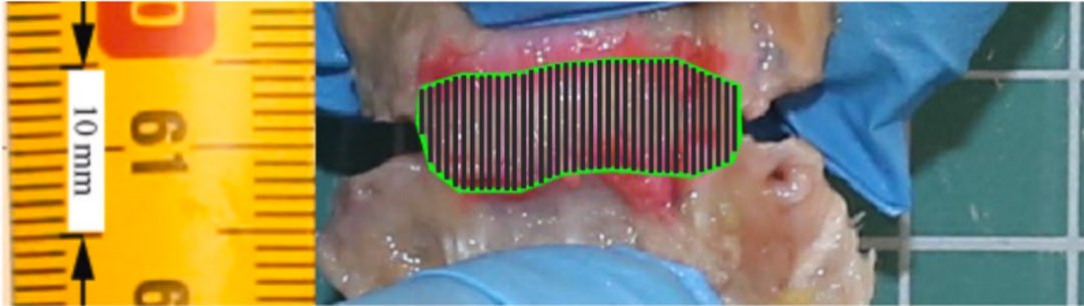
Determination of the initial length for ALL in a CAD environment

The boundaries of the ligaments were identified by an anatomy specialist through visual assessment of tissue texture, a tactile evaluation of differences in stiffness, and an analysis of the displacement range during the rotation of bone fragments. The initial length $\:l$ of the ligament was assumed as the average distance between the bone attachments. To determine this, the boundaries of the ligaments were marked at the edge of the bone using a copying pencil, and the specimens were subjected to an initial preload of approximately 50 g. Next, photographs were taken from both sides of each specimen with the camera mounted on a tripod. The camera was aligned so that its optical axis was as perpendicular as possible to the background reference ruler. These photographic images were then uploaded to a computer-aided design environment (AutoCAD, Autodesk, Inc., USA) for further analysis. The images were scaled to their true size using a ruler with 1 mm divisions. The midlines of the marks were drawn with polylines whose width was set to 0. The boundary of the tested ligament is indicated by the green line in Fig. 2. A vertical array of black lines was drawn at 0.5 mm intervals, and the distance between boundaries was measured along each line. The initial length $\:l$ was subsequently defined as the mean distance averaged across all such lines with 0.1 mm precision and was used to calculate the specimens’ stretch $\:\lambda\:$. Following the approach described in reference^10^, the cross-sectional area $\:A$ of each sample was estimated using the length-to-area ratio *r*. The ratios *r* for all ligaments were determined based on the mean values from the study^7^. For the LFs, and CLs, the relevant length corresponds directly to the initial length $\:l$. However, for the ALL and PLL, the relevant length is defined as the distance between the centers of adjacent vertebrae. For these ligaments, the centers of the vertebrae were identified as the midpoint of the distance between the ligament attachments to the vertebrae, and the inter-centroid distance was subsequently measured within the graphical software^7,8,10^.

#### Mounting to the testing machine

**Fig. 3:**
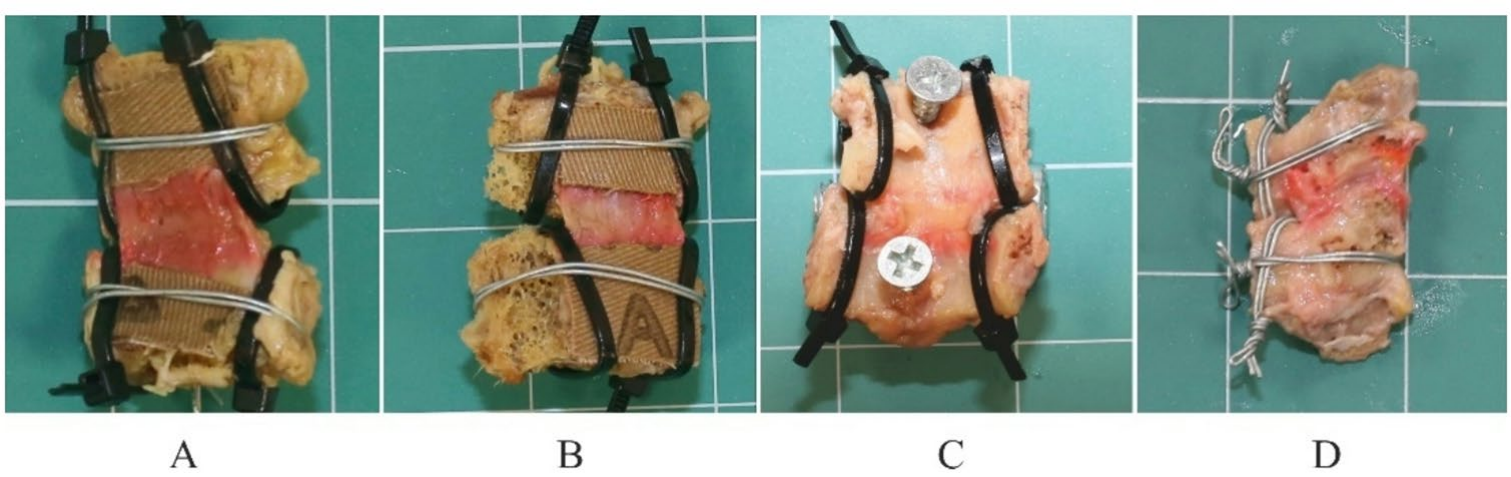
Reinforcement of bone parts. (**A**) ALL - front view, (**B**) PLL - front view, (**C**) LF - front view, and (**D**) CL – side view

The samples were removed from the freezer and placed in saline for approximately one hour. To mount the BLB samples in the testing machine, the specimens were placed into 3D-printed PLA cups^24^ and potted using resin (Technovit 3040, Kulzer GmbH) mixed with sand (CEN EN 196-1 standard)^25^. To enhance friction between the bone and the resin and to mitigate damage associated with boundary effects, the BLB complexes were reinforced as shown in Fig. 3. For ALL and PLL, sandpaper was applied to improve grip. Additionally, two cable ties were used to compress the vertebral body longitudinally, and circumferential wire wrapping was used for further stabilization (see Fig. 3A and B). For the LF, a centrally positioned steel screw was inserted into the middle of the bone (Fig. 3C), along with longitudinal reinforcement using cable ties. The CL BLB complexes were reinforced both circumferentially and longitudinally using wire ties (see Fig. 3D).

The casting of the reinforced samples was conducted as follows: for the ALL and the PLL samples, the cups were filled up to the level of the ligament attachment. For the LF, the cups were filled to completely cover the centrally positioned screw. Similarly, for the CL, the resin mixture was added to cover all applied reinforcement. During the curing process, the samples were wrapped in saline-soaked lignin to maintain moisture. After 15 min, the opposite side of each sample was mounted following the same procedure. Once both sides were prepared, the samples were wrapped in several layers of plastic stretch film and stored in a refrigerator at approximately 5 °C for 24 h to ensure complete curing.

### Tensile tests

#### Low-strain rate

**Fig. 4:**
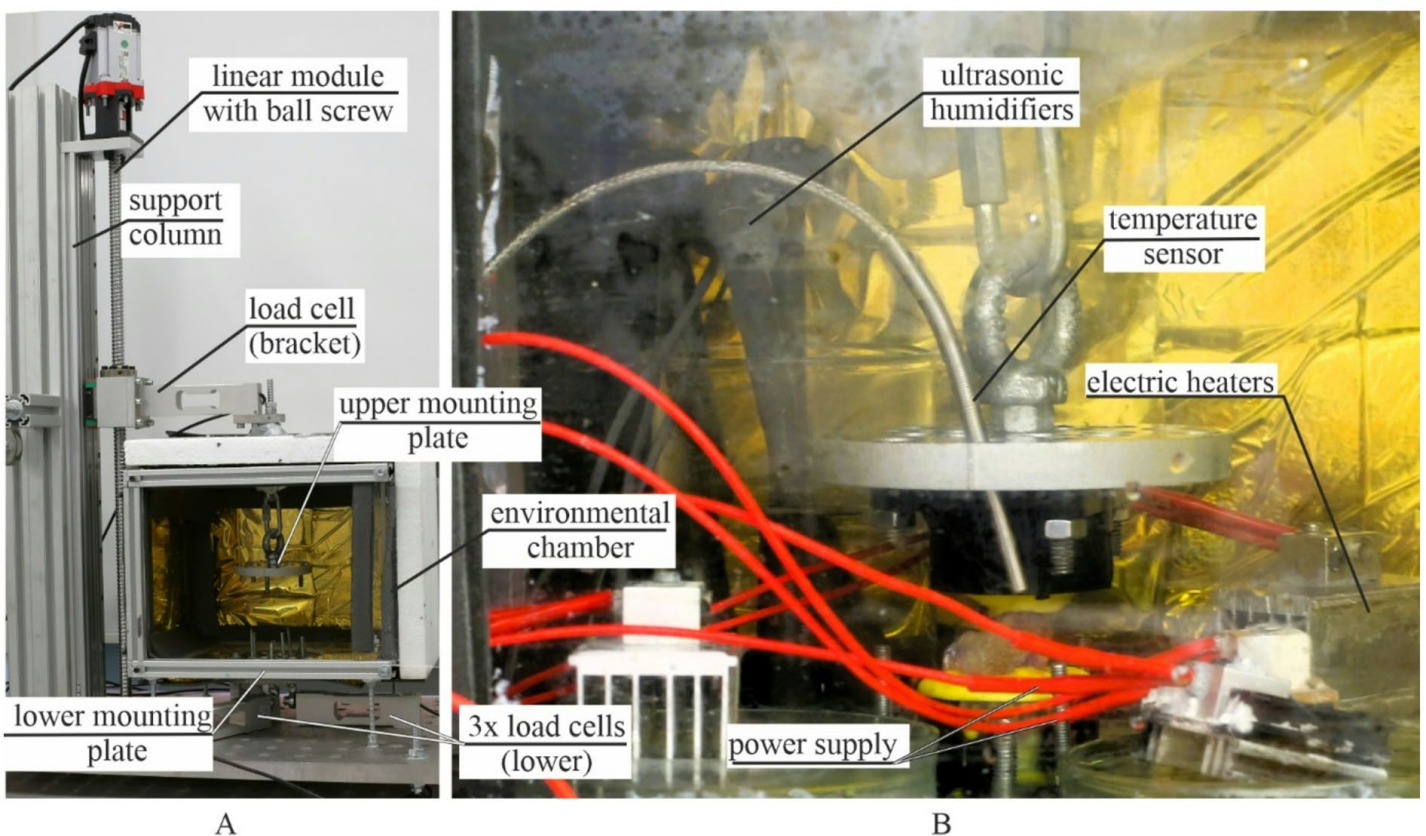
Experimental setup for low-strain rate test: (**A**) custom-made machine (**B**) environmental chamber

Before testing, the samples were removed from the refrigerator and immersed in physiological saline solution. Then samples were mounted in the tensile testing machine and subjected to an initial preload of 5 N tensile force, applied at a strain rate of 0.5 s⁻¹^10^. Subsequently, the samples were allowed to relax for 60 s^26^. Then, preconditioning was performed, which involved 60 cycles of 10% strain ($\:0.1l$) at a frequency of 1 Hz, followed by a 60 s recovery period. During the tensile testing phase, the samples were continuously strained at a rate of 0.5 s⁻¹ until complete rupture. All tests were conducted in an environmental chamber (see Fig. 4B) maintained at a constant temperature of 36.6 °C and a minimum relative humidity of 95%.

The tests were carried out using a custom-designed machine (Patent application: P.443948) (see Fig. 4A). This device features an AC stepper motor (Leadshine ELM-0400LH60F-SS-400 W) controlled by a servo controller (Leadshine ELP-RS400Z, 0.001 mm accuracy), which is connected to a linear module (YR-HGWS60K SFU1605). Force data were acquired from three bottom-mounted load cells (Utilcell M140, up to 1500 N). The displacement of the load cell (bracket) was recorded using a servo controller and a universal amplifier HBM QuantumX MX840A (Hottinger Baldwin Messtechnik, Germany) with Catman Data Acquisition Software.

#### High-strain rate

**Fig. 5:**
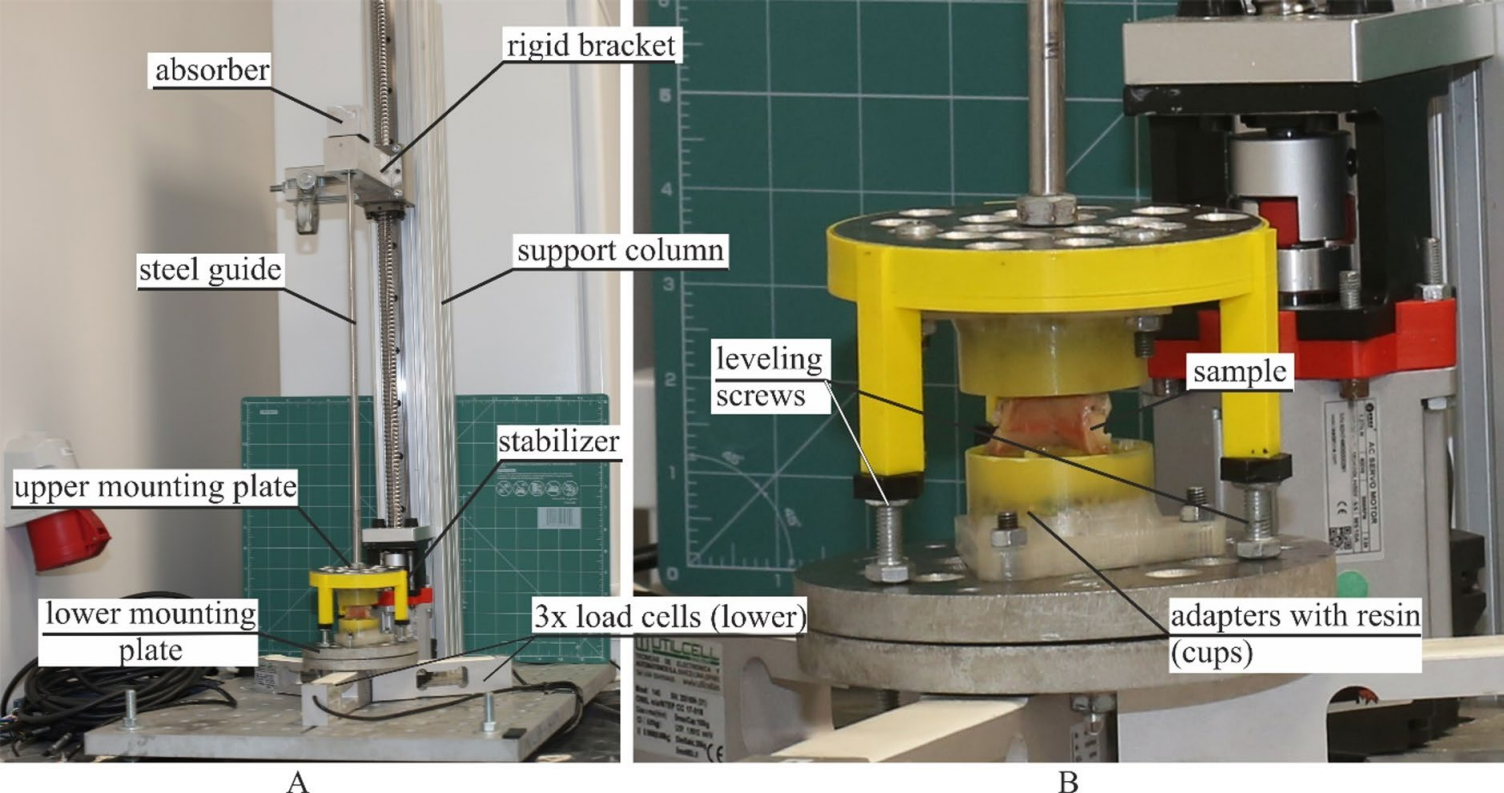
Experimental setup for high-strain rate test: (**A**) custom-made machine (**B**) sample view

The high-strain rate tests were conducted using a modified version of the custom-designed tensile testing machine described in the previous section (see Fig. 5). The top-mounted load cell (bracket) was replaced with a rigid bracket. A steel guide with an absorber was installed through the hole in the rigid bracket. Preconditioning and data acquisition were carried out in the same way as described in the low-strain rate test section. To avoid eccentric position of the sample, a 3D-printed stabilizer with leveling screws was used. During the high-strain rate tensile test, the machine accelerated the rigid bracket to a strain rate of 50 s⁻¹ and maintained this value until the sample completely ruptured. The test configuration required a modification of the environmental control procedure. Instead of an environmental chamber, the specimens were stored in physiological saline solution at 36.6 °C immediately prior to testing. The entire main test was conducted within 10 min of removing the specimen from the solution.

#### Force-displacement post-processing

**Fig. 6:**
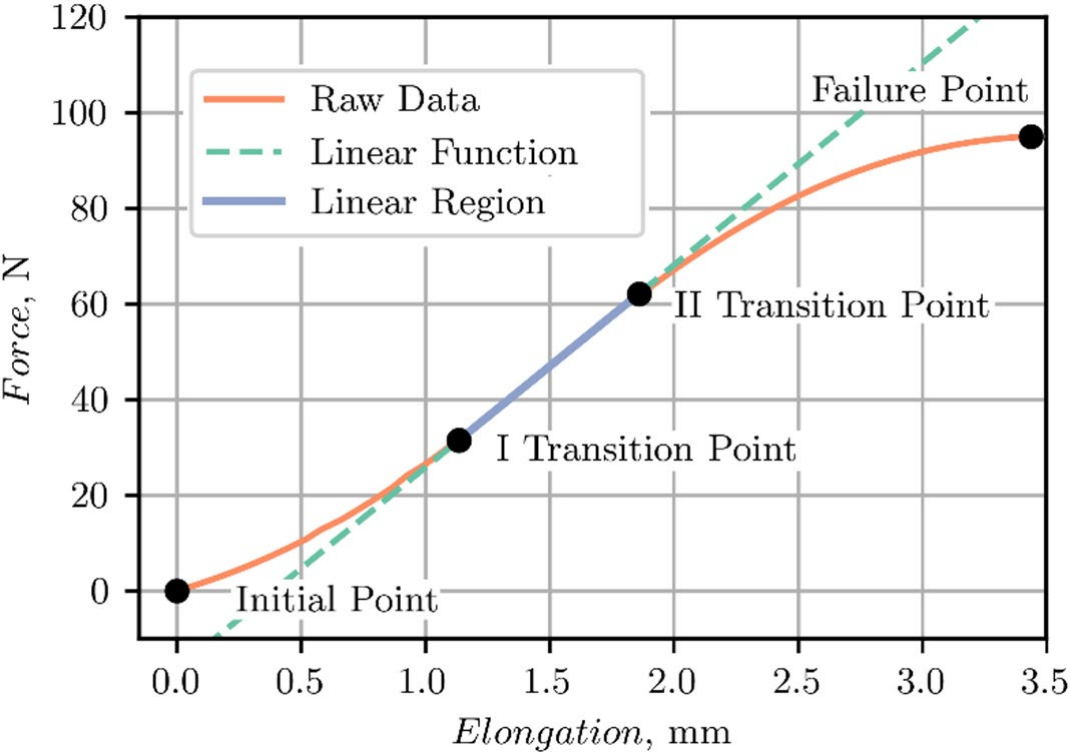
Typical points of force-elongation curve

The post-processing steps described below were carried out in a custom Python script. A standardized post-processing procedure was implemented to extract characteristic regions and points from low-strain and high-strain rate ligament tensile tests. The raw force-displacement curves were segmented based on the well-established three-phase response of ligamentous tissue: (1) the toe region (from the initial point to the I transition point), (2) the linear region (between the I and II transition point), and (3) the sub-failure region (spanning from the II transition point to the failure point) (see Fig. 6)^11,27^. These points (initial, I transition, II transition, and the failure points) were systematically identified using a multi-step computational method, summarized as follows.

The raw data (displacement $\:\varDelta\:u$ and force $\:F$) were smoothed with Savitzky–Golay filters to obtain a stable derivative of the force signal. An onset index of relevant data was established by requiring that both the smoothed derivative and the force exceed specified thresholds for a persistent window of samples. Additional points before the onset index were included in the clipped data to retain enough data for subsequent filtering. Then, the load-displacement curve was trimmed slightly beyond the peak force to ensure that the clipped data included the full ligament test.

Next, the clipped data were processed using a fifth-order, two-pole, Butterworth low-pass filter and an adaptive cutoff frequency selection approach. Cutoff frequencies were determined by integrating the Energy Spectral Density of the force signal until 95% of the energy was captured^28^. Additionally, a maximum cutoff frequency of 300 Hz was adopted to consider the physiological limitations of wave propagation in soft tissues based on SAEJ211 guidelines^29^. This hybrid approach suppressed measurement noise, without attenuating the relevant mechanical response. After filtering, indices corresponding to the initial point and failure point were identified in near-zero derivative regions to eliminate redundant data associated with machine stabilisation or late-stage noise.

The linear region for identifying the I and II transition points (see Fig. 6) was found by a binary search that located the largest segment exhibiting a linear fit (adjusted $\:R^{2}>99.5\%$) above a threshold. The segments from early toe and later sub-failure regions were excluded by imposing displacement bounds.

#### Averaging of experimental curves

The displacement increment $\:\varDelta\:u$ was used to calculate each specimen stretch value with the following formula $\:\lambda\:=\frac{l+\varDelta\:u}{l}$, where $\:l$ is sample’s initial length. To obtain the corresponding stress value $\:P=\frac{F}{A}$ (the engineering stress), force $\:F$ value was divided by sample’s initial cross-sectional area $\:A$. Characteristic points (I and II transition points) on the stress-stretch curves were averaged to obtain average ligament curves up to the averaged II transition points. Then, a linear function was fitted between the averaged I and II transition points, whereas the average tissue response between the initial point and the I transition point was captured by an exponential convex function, maintaining *C*¹ continuity with the linear function and ensuring a non-decreasing trend.

### Transversely isotropic visco-hyperelastic constitutive model

The ligament structure is conceptualized as a composite material consisting of a base extracellular matrix strengthened by embedded collagen fibers.

The hyperelastic potential function, $\:{\text{W}}_{\text{i}\text{s}\text{o}}^{\text{e}}$ is split into the matrix $\:{\text{W}}_{\text{m}}^{\text{e}}$ and fibers $\:{\text{W}}_{\text{f}}^{\text{e}}$ parts^1,30^:

1 $$\:{\text{W}}_{\text{i}\text{s}\text{o}}^{\text{e}}\left({\stackrel{-}{I}}_{1},J,\:{\stackrel{-}{I}}_{4}\right)={\text{W}}_{\text{m}}^{\text{e}}\left({\stackrel{-}{I}}_{1},J\right)+{\text{W}}_{\text{f}}^{\text{e}}\left({\stackrel{-}{I}}_{4}\right),$$

where $\:J=det\left(\mathbf{F}\right)$ is the Jacobian of deformation gradient $\:\mathbf{F}$, variables $\:\stackrel{-}{{I}_{1}}=\text{t}\text{r}\left(\stackrel{-}{\mathbf{C}}\right)$, $\:\stackrel{-}{{I}_{4}}={\mathbf{A}}_{0}\hspace{0.17em}:\hspace{0.17em}\stackrel{-}{\mathbf{C}}$ are invariants of right Cauchy-Green tensor $\:\stackrel{-}{\mathbf{C}}={\stackrel{-}{\mathbf{F}}}^{\text{T}}\stackrel{-}{\mathbf{F}}$, in which $\:\stackrel{-}{\mathbf{F}}={J}^{-\frac{1}{3}}\mathbf{F}$, and $\:{\mathbf{A}}_{0}={\mathbf{a}}_{0}\otimes\:{\mathbf{a}}_{0}$ is the fiber direction tensor in initial configuration.

A Neo-Hookean model was used to describe the matrix part $\:{\text{W}}_{\text{m}}^{\text{e}}$^31^ with volumetric term suggested by Martins et al.^32^, resulting in:

2 $$\:{\text{W}}_{\text{m}}^{\text{e}}({\stackrel{-}{I}}_{1},J)={C}_{10}\left(\stackrel{-}{{I}_{1}}-3\right)+\frac{1}{{D}_{1}}{\left(J-1\right)}^{2},$$

where $\:{C}_{10}=0.5\mu\:$, $\:\mu\:$ is a shear modulus in MPa, $\:{D}_{1}=\frac{2}{K}$ is a compressibility constant in $\:\text{M}\text{P}{\text{a}}^{-1}$ and $\:K$ in MPa represents the bulk stiffness^33^. For incompressible materials, $\:{D}_{1}$ can be also calculated using:

3 $$\:{D}_{1}=\frac{3(1-2\nu\:)}{\mu\:\left(1+\nu\:\right)},$$

where *v* is the Poisson ratio.

The polynomial function capturing the behavior of the fibers is expressed as:

4 $$\:{\text{W}}_{\text{f}}^{\text{e}}\left(\stackrel{-}{{I}_{4}}\right)=\left\{\begin{array}{c}0,\:\:\:\:\:\:\:\:\:\:\:\:\:\:\:\:\:\:\:\:\:\:\:\:\:\:\:\:\:\:\:\:\:\:\:\:\:\:\:\:\:\:\:\:\:\:\\\:{C}_{2}{\left(\stackrel{-}{{I}_{4}}-1\right)}^{2}+{C}_{3}{\left(\stackrel{-}{{I}_{4}}-1\right)}^{4},\end{array}\right.\quad\begin{array}{c}\stackrel{-}{{I}_{4}}\le\:1\\\:\stackrel{-}{{I}_{4}}>1\end{array}$$

The viscous isochoric component $\:{{\uppsi\:}}_{\text{i}\text{s}\text{o}}^{\text{v}}$ is associated with both the matrix, and the fibers, characterized by the material constants $\:{\eta\:}_{1}$, $\:{\eta\:}_{2}$, respectively. The contributions from these terms are added according to the method proposed by^30,34,35^:

5 $$\:{{\uppsi\:}}_{\text{i}\text{s}\text{o}}^{\text{v}}\left(\stackrel{-}{{I}_{1}},\stackrel{-}{{J}_{2}},\stackrel{-}{{I}_{4}},\stackrel{-}{{J}_{5}}\right)={{\uppsi\:}}_{\text{m}}^{\text{v}}\left(\stackrel{-}{{I}_{1}},\stackrel{-}{{J}_{2}}\right)+{{\uppsi\:}}_{\text{f}}^{\text{v}}\left(\stackrel{-}{{I}_{4}},\stackrel{-}{{J}_{5}}\right),$$

6 $$\:{{\uppsi\:}}_{\text{i}\text{s}\text{o}}^{\text{v}}\left(\stackrel{-}{{I}_{1}},\stackrel{-}{{J}_{2}},\stackrel{-}{{I}_{4}},\stackrel{-}{{J}_{5}}\right)=\left\{\begin{array}{c}{\eta\:}_{1}\stackrel{-}{{J}_{2}}\left(\stackrel{-}{{I}_{1}}-3\right),\:\:\:\:\:\:\:\:\:\:\:\:\:\:\:\:\:\:\:\:\:\:\:\:\:\:\:\:\:\:\:\:\:\:\\\:{\eta\:}_{1}\stackrel{-}{{J}_{2}}\left(\stackrel{-}{{I}_{1}}-3\right)+\frac{1}{2}{\eta\:}_{2}\stackrel{-}{{J}_{5}}{\left(\stackrel{-}{{I}_{4}}-1\right)}^{2},\end{array}\right.\begin{array}{c}\stackrel{-}{{I}_{4}}\le\:1\\\:\stackrel{-}{{I}_{4}}>1\end{array}$$

where $\:\stackrel{-}{{J}_{2}}=\frac{1}{2}\text{t}\text{r}\left({\dot{\stackrel{-}{\mathbf{C}}}}^{2}\right)\hspace{0.17em}$ and $\:\stackrel{-}{{J}_{5}}={\mathbf{A}}_{0}\hspace{0.17em}:\hspace{0.17em}{\dot{\stackrel{-}{\mathbf{C}}}}^{2}$ are viscous invariants of derivative of the right Cauchy-Green tensor $\:\dot{\stackrel{-}{\mathbf{C}}}={\dot{\stackrel{-}{\mathbf{F}}}}^{\text{T}}\hspace{0.17em}\stackrel{-}{\mathbf{F}}+{\stackrel{-}{\mathbf{F}}}^{\text{T}}\hspace{0.17em}\dot{\stackrel{-}{\mathbf{F}}}$. The terms are influenced by the square of $\:\dot{\stackrel{-}{\mathbf{C}}}$, guaranteeing a dissipation potential that is positive-definite and responds to the magnitude of the strain rate, independent of its direction^30,35^.

#### Material parameters identification for uniaxial stress state

For the considered material model, five unique material parameters ($\:{C}_{10}$, $\:{C}_{2}$, $\:{C}_{3}$, $\:{\eta\:}_{1}$ and $\:{\eta\:}_{2}$) must be established. In the present study they are identified via curve-fitting procedure by making use of the experimental data from uniaxial tensile tests up to II transition point. Denoting the displacement direction as 1, which is the fibers direction, the uniaxial stress state expressed in terms of engineering stress $\:{P}_{11}$ yields $\:{P}_{11}\ge\:0,\:{P}_{22}={P}_{33}=0$. Assuming material incompressibility $\:\left(\nu\:\approx\:0.5,J\equiv\:1,\:{\lambda\:}_{1}=\lambda\:,{\lambda\:}_{2}={\lambda\:}_{3}=\frac{1}{\sqrt{\lambda\:}}\right)$ the invariants defining the energy function read:

7 $$\:{I}_{1}={\lambda\:}^{2}+\frac{2}{\lambda\:},\:{I}_{4}={\lambda\:}^{2},\:\:$$

8 $$\:{J}_{2}={\dot{\lambda\:}}^{2}\left(2{\lambda\:}^{2}+\frac{1}{{\lambda\:}^{4}}\right),{J}_{5}=4{\dot{\lambda\:}}^{2}{\lambda\:}^{2}.$$

The first principal value of the engineering stress $\:{P}_{11}$, which is used for fitting procedures can be obtained from the following chain rule^36^:

9 $$\:{P}_{11}=\frac{\partial\:{\text{W}}_{\text{i}\text{s}\text{o}}^{\text{e}}}{\partial\:{I}_{1}}\frac{\partial\:{I}_{1}}{\partial\:\lambda\:}+\frac{\partial\:{\text{W}}_{\text{i}\text{s}\text{o}}^{\text{e}}}{\partial\:{I}_{4}}\frac{d{I}_{4}}{d\lambda\:}+\frac{\partial\:{{\uppsi\:}}_{\text{i}\text{s}\text{o}}^{\text{v}}}{\partial\:{J}_{2}}\frac{\partial\:{J}_{2}}{\partial\:\dot{\lambda\:}}+\frac{\partial\:{{\uppsi\:}}_{\text{i}\text{s}\text{o}}^{\text{v}}}{\partial\:{J}_{5}}\frac{\partial\:{J}_{5}}{\partial\:\dot{\lambda\:}}.$$

Finally, using Eq. (9) together with the energy functions Eqs. (2), (4), **(6)** and Eqs. (7), (8), the expression for $\:{P}_{11}$ is obtained as follows:

10 $$\begin{aligned}\:{P}_{11}=&\:2\lambda\:\left[{C}_{10}\left(1-\frac{1}{{\lambda\:}^{3}}\right)+2{C}_{2}\left({\lambda\:}^{2}-1\right)+4{C}_{3}{\left({\lambda\:}^{2}-1\right)}^{3}\right]\\&\quad+2\dot{\lambda\:}\left[{\eta\:}_{1}\left(2{\lambda\:}^{2}+\frac{1}{{\lambda\:}^{4}}\right)\left({\lambda\:}^{2}+\frac{2}{\lambda\:}-3\right)+2{\eta\:}_{2}\hspace{0.17em}{\lambda\:}^{2}{\left({\lambda\:}^{2}-1\right)}^{2}\right].\end{aligned}$$

The material constants were determined by fitting the equation (Eq. (10)) through a nonlinear least-squares method (Python’s *scipy.optimize* library) to the experimental data up to the II transition point stretch value ($\:{\lambda\:}_{\text{I}\text{I}}$).

While fitting the (Eq. (10)) to the low-strain-rate data (0.5 s⁻¹) the assumption of negligible viscous effects ($\:{\eta\:}_{1},\:{\eta\:}_{2}\approx\:0$) was made, leading to the determination of three unique material constants ($\:{C}_{10}$, $\:{C}_{2}$, and $\:{C}_{3}$). Additionally following constraints were made:

1. **Bounds**: The shear modulus was assumed to be $\:\mu\:\in\:[0.2;2.5]\hspace{0.17em}\text{MPa}$, implying $\:{C}_{10}\in\:[0.1;1.25]\hspace{0.17em}\:\text{MPa}$ consistent with range reported in the literature^1,35^ and basing on the authors’ experience that lower values than $\:0.2\hspace{0.17em}\text{MPa}$ of shear modulus may lead to numerical instabilities in FEM simulations,
2. **Convexity Condition**: $\:{C}_{10}$, $\:{C}_{2}$ and $\:{C}_{3}$ to be positive, ensuring the convexity of the energy functions,
3. **Energy Ratio Penalty**: a penalty term to enforce $\:\frac{{\text{W}}_{\text{m}}^{\text{e}}}{{\text{W}}_{\text{i}\text{s}\text{o}}^{\text{e}}}<0.5$ at the second transition point, to maintain a fiber-dominated response at higher stretches^1,27^. If this condition was satisfied, the penalty remained zero; otherwise, it was set to a high value (e.g., 100).

For the high-strain (50 s⁻¹) rate data viscous effects were included in the model, resulting in a set of five constants ($\:{C}_{10}$, $\:{C}_{2}$, $\:{C}_{3}$, $\:{\eta\:}_{1}$ and $\:{\eta\:}_{2}$). The following assumptions were implemented during the curve‑fitting procedure:

1. **Parameter Penalty**: A penalty term was introduced to ensure that the mean values of $\:{C}_{10}$, $\:{C}_{2}$, and $\:{C}_{3}$ remained close to those determined at the low-strain rate (0.5 s⁻¹).
2. **Bounds**: The minimum and maximum values of $\:{C}_{10}$, $\:{C}_{2}$, and $\:{C}_{3}$ derived from the low‑strain rate (0.5 s⁻¹) dataset were used as parameter bounds.
3. **Convexity Condition**: The parameters $\:{\eta\:}_{1}$ and $\:{\eta\:}_{2}$ were constrained to be positive, thereby maintaining the convexity of the energy functions.
4. **Energy Ratio Penalty**: Analogous to the low‑strain‑rate fitting, an additional penalty was imposed at the second transition point to enforce $\:\frac{{\text{W}}_{\text{m}}^{\text{e}}+{\text{W}}_{\text{m}}^{\text{v}}}{{\text{W}}_{\text{f}}^{\text{e}}+{\text{W}}_{\text{f}}^{\text{v}}}<0.5$, where.
5. $$\:{\text{W}}_{\text{m}}^{\text{v}}={\int\:}_{1}^{{\lambda\:}_{\text{I}\text{I}}}{P}_{11,m}^{\text{v}}\hspace{0.17em}\text{d}\lambda\:$$ and $$\:{\text{W}}_{\text{f}}^{\text{v}}={\int\:}_{1}^{{\lambda\:}_{\text{I}\text{I}}}{P}_{11,f}^{\text{v}}\hspace{0.17em}\text{d}\lambda\:$$.

The Adj. *R*^2^, normalized root mean square error (NRMSE), and normalized mean absolute error (NMAE) metrics were used to evaluate the agreement between the experimental data and the fitted model.

### Statistical analysis

Statistical analysis was performed using the *statsmodels* library in the Python environment^37^. In this analysis, the results of ligament stiffness, elastic modulus, failure force, and failure stress were compared under two loading conditions – low-strain and high-strain rate. For each ligament group, the mean and standard deviation were calculated, providing an initial assessment of the measurement characteristics. To evaluate the significance of differences between the loading conditions, statistical tests were applied: if the data exhibited a normal distribution (as confirmed by the Shapiro-Wilk test), a Welch’s t-test was performed; otherwise, the non-parametric Mann-Whitney U test was employed.

## Results

### Experimental results and post-processing

 The geometric and mechanical properties of four cervical spine ligaments under low‑strain and high‑strain loading conditions are presented in Table 2. The parameters include cross‑sectional area *A*, initial length *l*, stiffness (slope in the linear region of the *F*($\:\varDelta\:u$) curve), and elastic modulus *E* (slope in the linear region of the $\:{P}_{11}\left({{\uplambda\:}}_{1}\right)$ curve). Additionally, failure force *F*_*f*_ represents the load at ligament rupture, while failure stress corresponds to the $\:{P}_{11,f}$ value at failure. Transition points I and II, determined via the procedure outlined in Sect. 2.2.3, are reported in terms of the displacement ($\:\varDelta\:u$) and force at each point. The mean length of the cervical spine ligaments ranged from 6.4 mm to 9.9 mm, while the mean failure force *F*_*f*_ ranged from 118.6 N to 247.9 N. Among the ligaments examined, the highest modulus of elasticity was found in the posterior longitudinal ligament (PLL) (72.81 MPa at high-strain load), while the lowest value was found in the capsular ligament (CL) (8.59 MPa at low-strain load). Sample dimensions and mechanical properties of the ligaments, expressed as medians and quartiles, are provided in the Appendix.

**Table 2:** Geometric data and mechanical properties of ligaments expressed as the mean (± SD)

				I transition point		II transition point					
Lig.	Strain Rate	Area, mm^2^	Length, mm	d, mm	F, *N*	d, mm	F, *N*	Stiffness, *N*/mm	E, MPa	F_f_, *N*	$\:{\varvec{P}}_{11,\varvec{f}}$, MPa
**ALL**	Low	12.8 ± 2.3	6.4 ± 1.9	0.86 ± 0.54	41.4 ± 33.28	2.23 ± 0.66	170.15 ± 49.67	106.36 ± 22.05	52.48 ± 14.24	212.26 ± 71.90	16.37 ± 3.52
**ALL**	High	13.7 ± 2.3	7.8 ± 1.1	0.94 ± 0.42	45.28 ± 25.79	1.63 ± 0.56	103.16 ± 46.55	85.65 ± 28.84	50.39 ± 22.39	174.69 ± 69.11	12.86 ± 4.66
**PLL**	Low	13.9 ± 2.7	6.4 ± 1.5	0.95 ± 0.84	36.42 ± 26.24	2.16 ± 0.95	138.87 ± 68.26	85.06 ± 20.57	39.21 ± 10.34	151.29 ± 65.68	11.43 ± 5.52
**PLL**	High	14.3 ± 2.3	8.8 ± 1.6	1.28 ± 0.6	65.56 ± 41.77	2.03 ± 0.6	146.44 ± 55.13	116.56 ± 31.88	72.81 ± 25.17	247.89 ± 130.23	18.37 ± 11.32
**LF**	Low	49.9 ± 8.7	9.9 ± 1.6	1.63 ± 0.85	38.41 ± 19.18	2.7 ± 0.82	101.84 ± 27.57	65.96 ± 30.33	13.1 ± 5.67	135.27 ± 54.23	2.71 ± 1.03
**LF**	High	46.0 ± 6.9	8.9 ± 1.9	0.52 ± 0.24	25.71 ± 14.42	1.21 ± 0.3	107.83 ± 45.53	117.64 ± 31.36	22.95 ± 7.25	163.42 ± 50.11	3.58 ± 1
**CL**	Low	45.6 ± 8.4	6.9 ± 0.9	0.75 ± 0.69	16.35 ± 6.62	2.23 ± 1.08	90.4 ± 40.54	55.58 ± 22.05	8.59 ± 3.6	130.44 ± 52.38	2.85 ± 0.88
**CL**	High	47.3 ± 9.8	7.3 ± 1.1	0.75 ± 0.25	27.21 ± 8.78	1.43 ± 0.44	79.32 ± 29.9	82.34 ± 30.49	12.86 ± 5.02	118.6 ± 45.05	2.61 ± 1.21

### Statistical analysis

Statistical analysis of mechanical properties was performed using quasi-static loading conditions as the baseline. Along with the percent change, Table 3 lists the corresponding *p*-value and the effect size (Cohen’s *d* or Cliff’s *δ* in the nonparametric case). In the Table 3, N stands for the number of samples, whereas S denotes the low-strain rate test, and D the high-strain rate test. The percentage change was computed as $\:\varDelta\:=\frac{\text{(D}-S\text{)}}{S}\times\:100\%.$ Welch’s t-test was applied in all comparisons except for the failure force (*F*_*f*_) in the CL group, where Mann–Whitney’s U test was chosen due to non‐normal data. A statistically significant effect of strain rate on stiffness and elastic modulus (marked with an asterisk) was identified in the PLL, LF, and CL groups. Specifically, stiffness and elastic modulus in the PLL group rose by 37.03% and 85.68%, respectively; in the LF group, they increased by 78.35% and 75.13%; and in the CL group, both parameters increased by 48.15% and 49.58%, respectively.

**Table 3:** The results of the statistical analysis of mechanical properties

Lig.	Mechanical Property	*N* S/D	$\:\varDelta\:$, %	*p*-value	Effect size
**ALL**	*Stiffness*, N/mm	6/9	−19.47	0.14	−0.78
	*E*, MPa	6/9	−3.98	0.83	−0.11
	*F*_*f*_, N	6/9	−17.70	0.34	−0.54
	$\:{P}_{11,f}$,MPa	6/9	−21.45	0.12	−0.82
**PLL**	*Stiffness*, N/mm	8/9	37.03	0.03*	1.16
	*E*, MPa	8/9	85.68	0.00*	1.71
	*F*_*f*_, N	8/9	63.86	0.07	0.92
	$\:{P}_{11,f}$, MPa	8/9	60.75	0.13	0.76
**LF**	*Stiffness*, N/mm	6/7	78.35	0.01*	1.67
	*E*, MPa	6/7	75.13	0.02*	1.50
	*F*_*f*_, N	6/7	20.81	0.36	0.54
	$\:{P}_{11,f}$, MPa	6/7	32.02	0.15	0.86
**CL**	*Stiffness*, N/mm	16/13	48.15	0.01*	1.02
	*E*, MPa	16/13	49.58	0.02*	0.99
	*F*_*f*_, N	16/13	−9.08	0.74	−0.08
	$\:{P}_{11,f}$, MPa	16/13	−8.40	0.56	−0.23

*statistically significant *p *< 0.05.

### Constitutive parameters

**Fig. 7:**
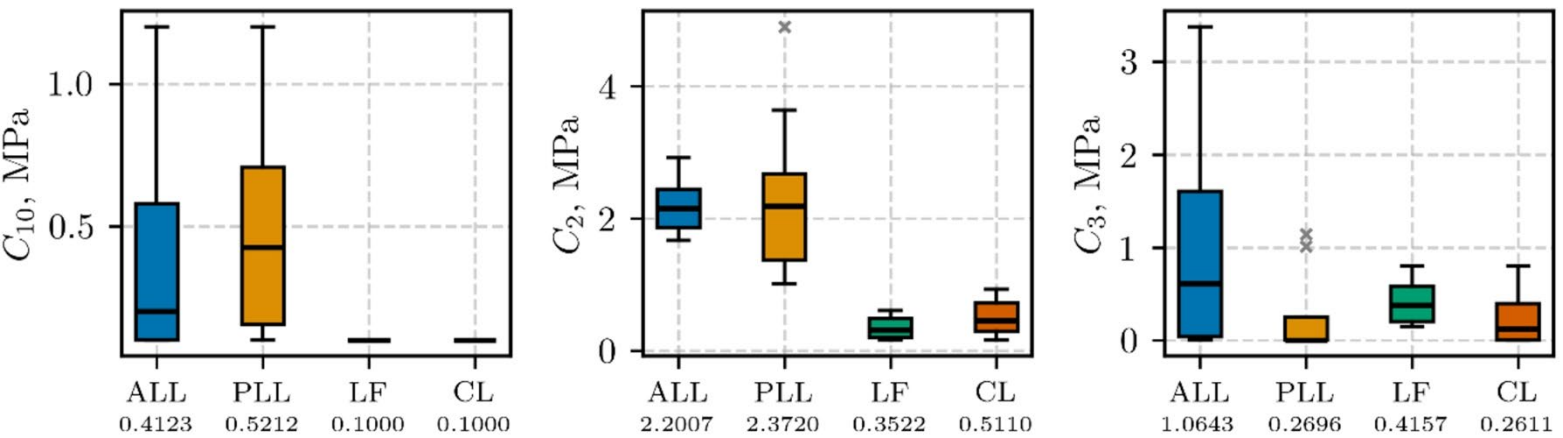
Boxplots of the hyperelastic parameters ($\:{C}_{10}$, $\:{C}_{2}$, and $\:{C}_{3}$) for $\:0.5\hspace{0.17em}{\text{s}}^{-1}$ experimental data

The boxplots of three hyperelastic parameters ($\:{C}_{10}$, $\:{C}_{2}$, and $\:{C}_{3}$) identified from low-strain rate tests (0.5 s⁻¹) are presented in Fig. 7 for each ligament type (ALL, PLL, LF, and CL). Each boxplot shows the inter-quartile range (box), median (horizontal line), and whiskers extending to 1.5 × IQR; outliers are indicated by grey $\:\times\:$ symbols. The mean value for each parameter appears beneath the corresponding ligament label.

The goodness-of-fit metrics (Adj. *R*², NRMSE, and NMAE) for the hyperelastic model fitted to experimental data for the low-strain rate tests are presented in Table 4. For all ligaments, the coefficient of determination *R²* exceeded 0.98, while the normalized root mean square error (NRMSE) and normalized mean absolute error (NMAE) remained low, ranging from approximately 0.02 to 0.03.

**Table 4:** Mean ± SD goodness-of-fit metrics for the hyperelastic model fitted to the low-strain rate data for individual specimens

Ligament	Adj. *R*²	NRMSE	NMAE
ALL	0.9928 ± 0.0064	0.0242 ± 0.0125	0.0198 ± 0.0099
PLL	0.9902 ± 0.0086	0.0278 ± 0.0117	0.0235 ± 0.0106
LF	0.9920 ± 0.0109	0.0225 ± 0.0167	0.0197 ± 0.0151
CL	0.9852 ± 0.0125	0.0367 ± 0.0163	0.0305 ± 0.0143

**Fig. 8:**
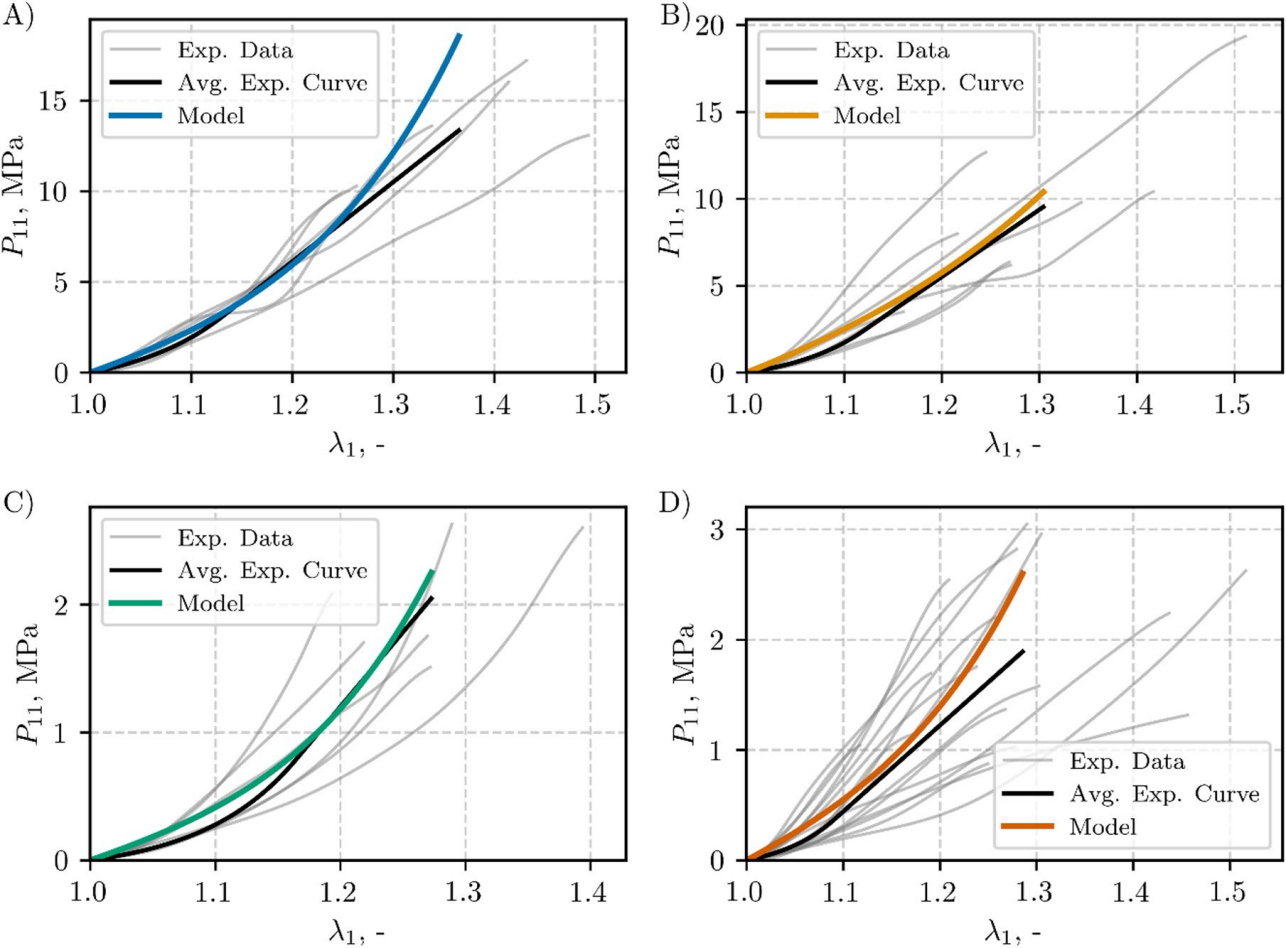
Hyperelastic mean-parameter model and experimental data, plotted up to the mean II transition stretch and the II transition stretch of the individual specimens, respectively: (**A**) ALL, (**B**) PLL, (**C**) LF, and (**D**) CL

The hyperelastic model obtained with the mean values of $\:{C}_{10}$, $\:{C}_{2}$, and $\:{C}_{3}$​ (see Fig. 7) was plotted up to each ligament’s mean II transition stretch value (see Fig. 8). Figure 8 also shows the corresponding experimental data (**Exp. Data**, grey lines), and averaged experimental data (**Avg. Exp. Curve**, black line) determined according to the method described in Sect. 2.2.4. The goodness-of-fit statistics (**Avg. Exp. Curve** vs. **Model**) are presented in Table 5. The coefficient of determination *R²* ranged from 0.69 for the capsular ligament (CL) to 0.96 for the ligamentum flavum (LF). The normalized root mean square error (NRMSE) varied between 0.05 and 0.16, while the normalized mean absolute error (NMAE) ranged from 0.04 to 0.14.

**Table 5:** Goodness-of-fit metrics for the mean-parameter hyperelastic model compared with the averaged experimental data for each ligament

Ligament	Adj. *R*²	NRMSE	NMAE
ALL	0.8323	0.1130	0.0963
PLL	0.8605	0.1063	0.0984
LF	0.9649	0.0516	0.0423
CL	0.6872	0.1561	0.1424

**Fig. 9:**
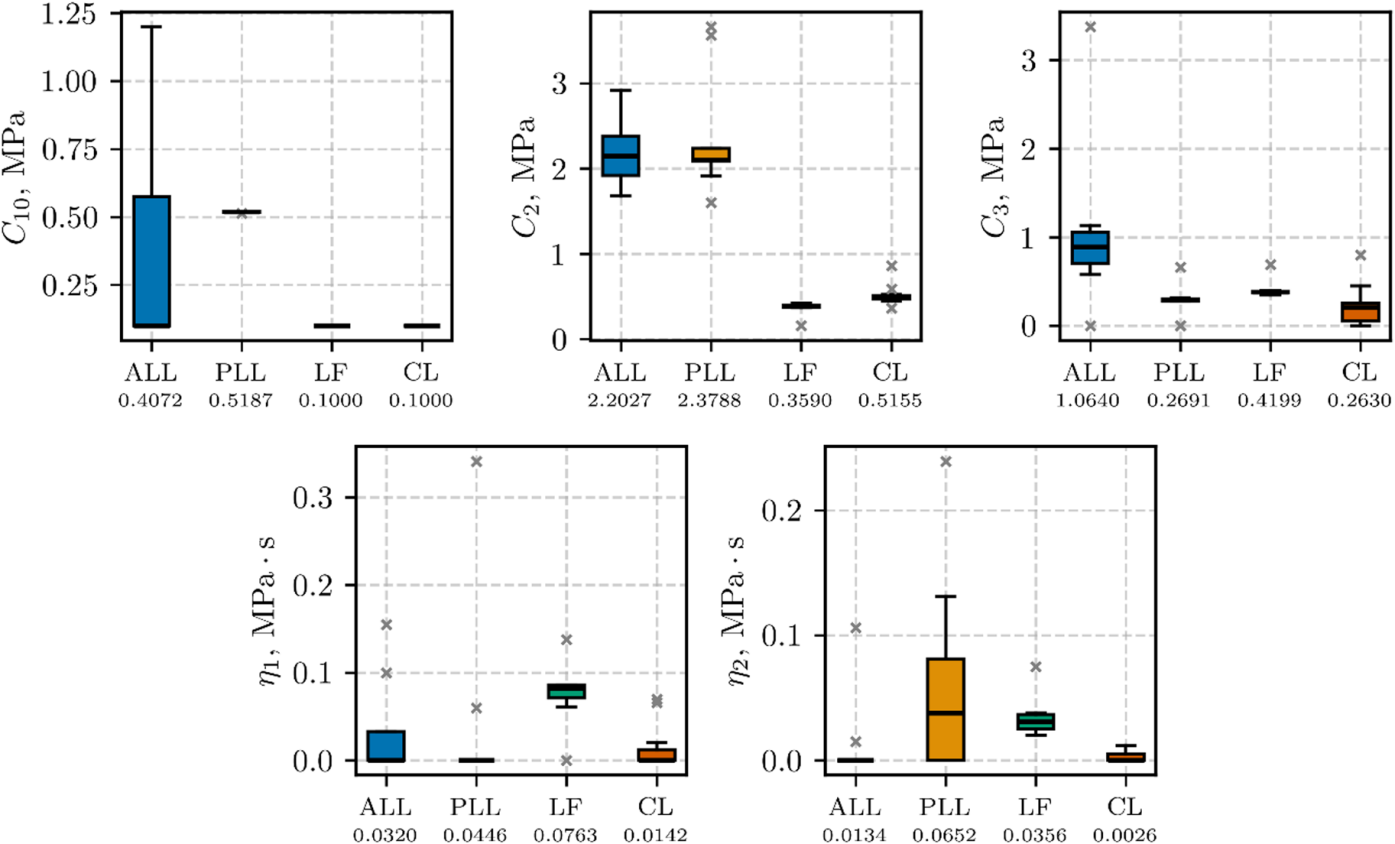
Boxplots of the visco-hyperelastic properties ($\:{C}_{10}$, $\:{C}_{2}$, $\:{C}_{3}$, $\:{\eta\:}_{1}$, and $\:{\eta\:}_{2}$) for $\:50\hspace{0.17em}{\text{s}}^{-1}$ experimental data

The boxplots of five parameters ($\:{C}_{10}$, $\:{C}_{2}$, $\:{C}_{3}$, $\:{\eta\:}_{1}$, and $\:{\eta\:}_{2}$) obtained from fitting the high-strain rate (50 s⁻¹) data are presented in Fig. 9, using the same boxplot conventions as described for Fig. 7. The highest median value of parameter $\:{\eta\:}_{1}$ was observed in the LF group, while the highest median value of parameter $\:{\eta\:}_{2}$ was found in the PLL group.

Table 6 presents the mean ± SD of the goodness-of-fit metrics (Adj. *R*², NRMSE, and NMAE) for the visco-hyperelastic model calibrated to experimental data for the high-strain rate tests. The coefficient of determination *R²* exceeded 0.96. The normalized root mean square error (NRMSE) varied between 0.02 and 0.04, while the normalized mean absolute error (NMAE) ranged from 0.02 to 0.04.

**Table 6:** Mean ± SD goodness-of-fit metrics for the visco-hyperelastic model fitted to the high-strain rate data for individual specimens

Ligament	Adj. *R*²	NRMSE	NMAE
ALL	0.9911 ± 0.0073	0.0234 ± 0.0116	0.0197 ± 0.0096
PLL	0.9782 ± 0.0158	0.0373 ± 0.0160	0.0313 ± 0.0130
LF	0.9719 ± 0.0206	0.0410 ± 0.0174	0.0321 ± 0.0132
CL	0.9643 ± 0.0481	0.0425 ± 0.0289	0.0358 ± 0.0256

**Fig. 10:**
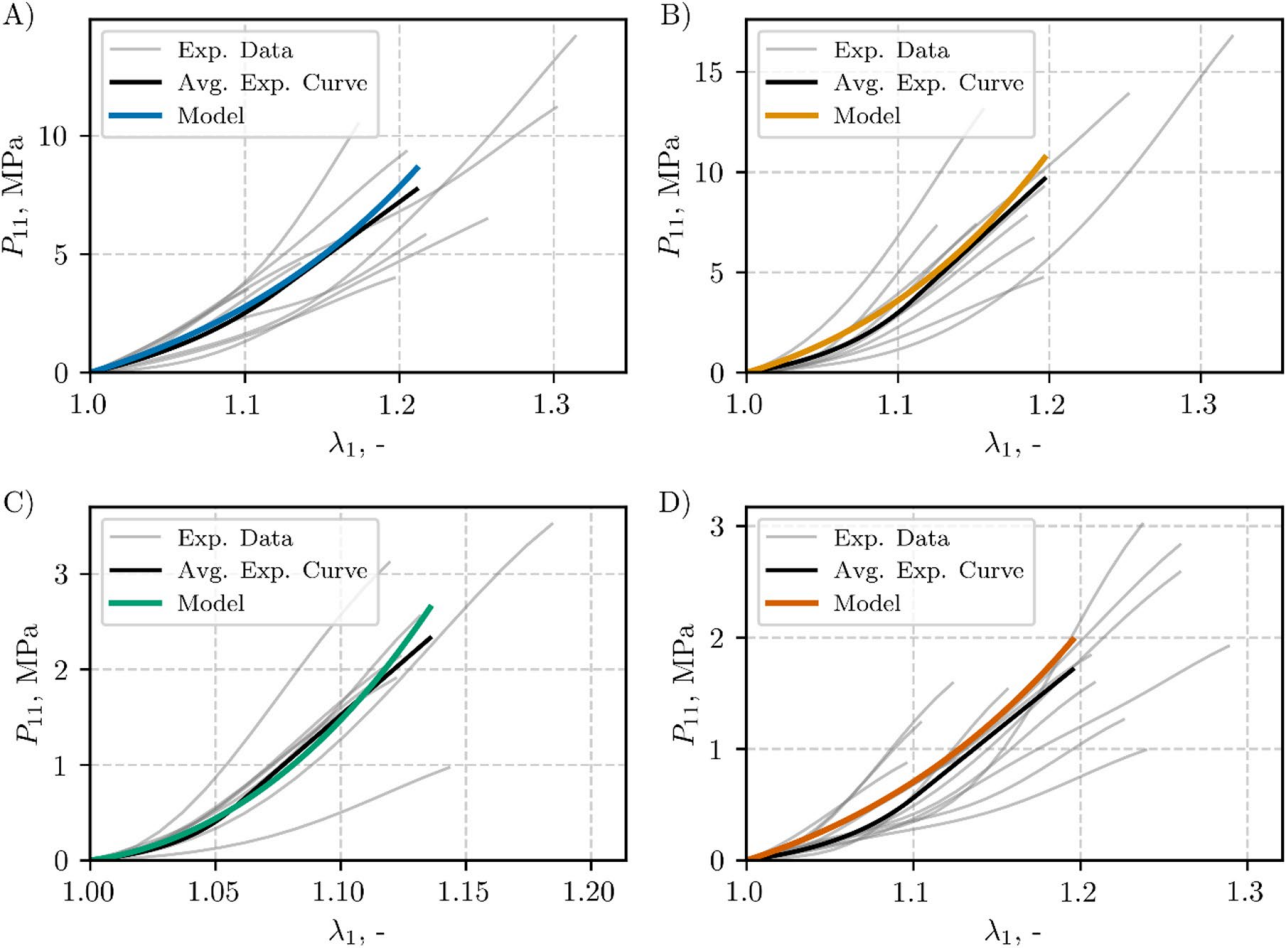
Visco-hyperelastic mean-parameter model and experimental data, plotted up to the mean II transition stretch and the II transition stretch of the individual specimens, respectively: (**A**) ALL, (**B**) PLL, (**C**) LF, and (**D**) CL

The visco-hyperelastic model was derived from the mean values of $\:{C}_{10}$, $\:{C}_{2}$, $\:{C}_{3}$, $\:{\eta\:}_{1}$, and $\:{\eta\:}_{2}$, as shown beneath the boxplots in Fig. 9. Figure 10 presents mean-parameters visco-hyperelastic model with the corresponding experimental data (**Exp. Data**, grey lines), and averaged experimental curve (**Avg. Exp. Curve**, black line), see Sect. 2.2.4. The goodness-of-fit statistics (**Avg. Exp. Curve** vs. **Model**) are summarized in Table 7. The coefficient of determination *R²* exceeded 0.96. The normalized root mean square error (NRMSE) varied between 0.03 and 0.07, while the normalized mean absolute error (NMAE) ranged from 0.03 to 0.06.

**Table 7:** Goodness-of-fit metrics for the mean-parameter visco-hyperelastic model compared with the averaged experimental data for each ligament

Ligament	Adj. *R*²	NRMSE	NMAE
ALL	0.9822	0.0408	0.0306
PLL	0.9890	0.0326	0.0250
LF	0.9653	0.0583	0.0508
CL	0.9588	0.0703	0.0640

## Discussion

Preliminary tests on animal samples revealed a critical issue related to the assembly and positioning of unreinforced BLB complexes in the testing apparatus. Specifically, the samples tended to slip out of the resin-sand mix, undermining the reliability and validity of the experimental data. Furthermore, when the unreinforced BLB complexes were stretched, the ligament attachment often detached, or the bone fractured, rather than experiencing ligament rupture. This failure mode was likely attributed to the inherent structural weakness of the ligament–bone interface or the brittleness of the bone when subjected to tension.

To address these challenges, the BLB complexes were reinforced to prevent undesirable damage such as bone fracture or bone–ligament separation during uniaxial tensile testing^2,10^. This reinforcement was necessary to ensure both sample integrity and the reliability of the test results^10,11^. As a result, no premature slippage occurred in any of the reinforced BLB complexes, and ultimate failure consistently occurred within the ligament tissue itself.

It should be acknowledged that the method used to estimate the cross-sectional area of the ligaments is approximate and may not yield fully accurate values of elastic modulus and failure stress. Direct measurement of the cross-sectional area requires destructive procedures, which are not feasible in the present context. In addition, despite cleaning, the specimens often contained surrounding tissues that could not be completely removed. Moreover, the inherently complex geometry of the ligaments makes non-invasive approaches are prone to measurement inaccuracies.

**Fig. 11:**
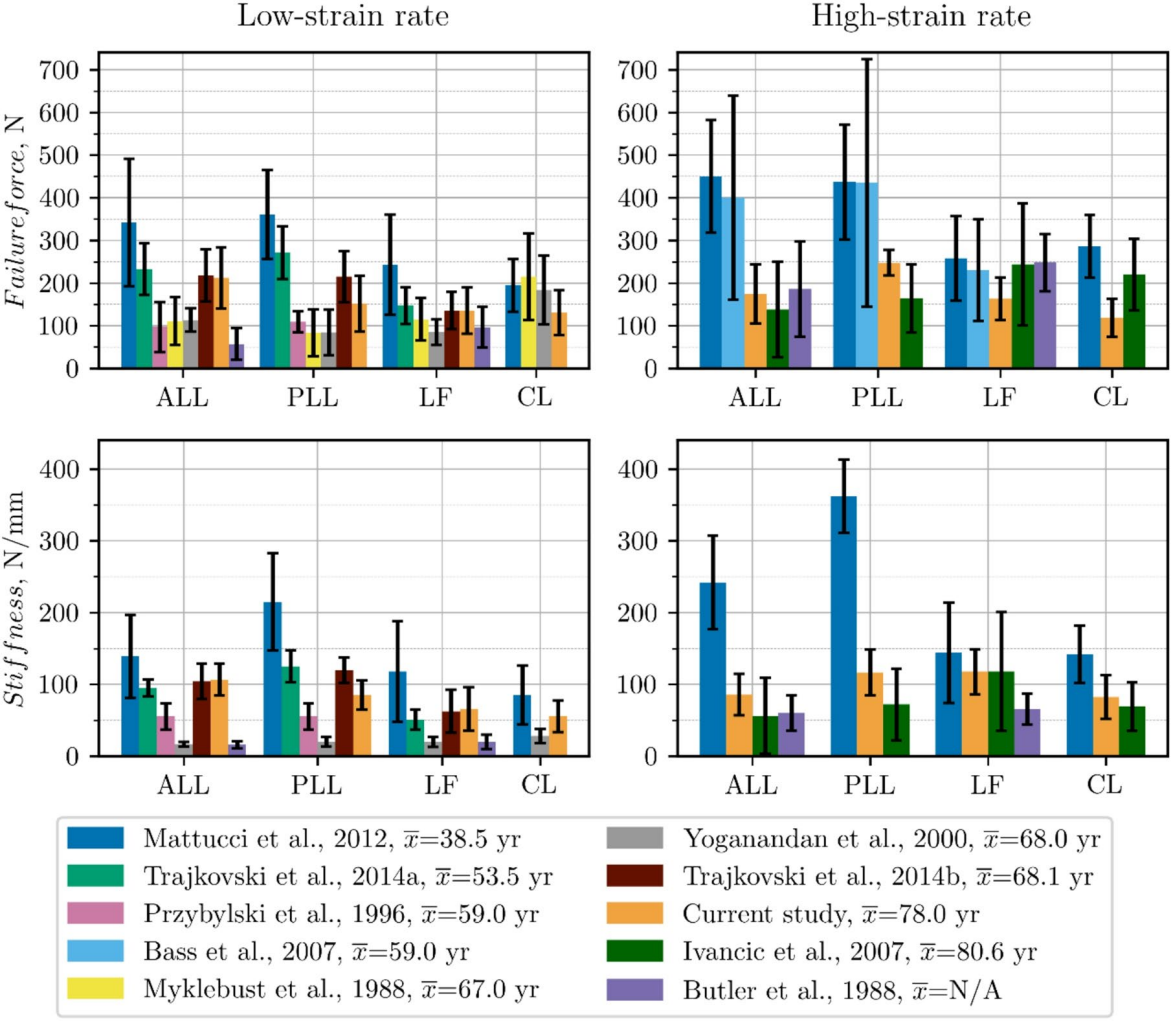
Comparison of failure force, and stiffness between current and literature experimental data

A comparison of ligamentous mechanical properties obtained in the current study with those from prior literature^1,5–12^ is illustrated in Fig. 11. The failure forces and stiffness values observed in the present study generally fall within the ranges reported in previous research. The observed discrepancies are likely due to differences in strain rate, donor age, sample preparation technique, demographic factors, and testing methodology. It is worth mentioning that despite the use of similar test conditions as the environmental chamber (for low-strain rate) and a standardized preconditioning protocol, the current mechanical properties were lower than those obtained in^10^. This can be explained by the fact that the average donor age (78 ± 6.34 years) in the present study was significantly higher than in^10^, where 27–50 years cadavers were studied. Furthermore, in^10^, the strain rate used in the high-strain rate tests was 3–5 times higher than in the current work. In general, the obtained values of failure forces and stiffness are in good agreement with the mechanical properties reported in studies involving older donors, such as^6,11^ for low-strain rate testing and^9^ for the high-strain rate testing. This is particularly evident for the LF ligament, for which the mean failure force in the present study (135.27 ± 54.23 N at 0.5 s⁻¹) is within the range (118.42 ± 27.48 N) obtained in^11^. Such consistency suggests that the mechanical properties of ligaments from older specimens converge to similar ranges under comparable testing conditions.

Younger donor populations often present ligaments with better-preserved collagen fiber orientations and less degenerative changes, thereby resulting in comparatively higher failure loads and greater stiffness^11,38^. This difference in tissue properties between younger and older populations underscores the importance of considering donor age and associated tissue quality when interpreting biomechanical data. Extrapolating biomechanical properties derived from younger cadaveric specimens to older populations risks overlooking the complex, age-related deterioration of the ligamentous tissues^39,40^. In the design of spinal implants, planning surgical stabilization procedures, evaluating dynamic events like sport accidents or car crashes, acknowledging these age-related differences is essential. Degenerative changes in the vertebrae and intervertebral joints may contribute to weakening of the ligament-bone interface, thereby reducing the overall load-bearing capacity and energy absorption potential of the vertebral column^41–43^, increasing the risk of injury and impaired function^39,40^.

A Weighted Least Squares (WLS) Meta-Regression was performed on data presented in Fig. 11. The effect of age on stiffness and failure force parameters for each ligament type was studied. High-strain and low-strain rates were considered separately. In each case, a negative regression coefficient value was obtained. However, the statistical significance and effectiveness of the regression model of this relationship were demonstrated only for the cases shown in Table 8. For the other cases, the proposed model did not perform well enough, and the *p*-value was greater than 0.05. The reason could be an insufficient amount of data.

The negative coefficients in Table 8 suggest that specimens harvested from significantly older donors have reduced stiffness if compared to cadaveric specimens from younger donors^44,45^. Over the human lifespan, the ligamentous tissues, comprising collagen and elastin, undergo progressive modifications at both the micro- and macrostructural levels, which are further intensified in the course of ageing^46,47^. Cumulative microdamage, such as microscopic tears and disruptions, along with collagen fiber disorganization and the infiltration of fibrotic and sclerotic processes, can render the tissue more fragile.

**Table 8:** Results of meta-regression

Ligament	Strain rate	Mechanical prop.	Adj. *R*^2^	*p*-value	Coef.
**ALL**	Low	*stiffness*	0.73	0.01	−1.88
**ALL**	High	*failure force*	0.88	0.04	−7.46
**PLL**	High	*stiffness*	0.99	0.05	−6.53
**LF**	High	*stiffness*	0.99	0.03	−0.66
**CL**	High	*stiffness*	0.98	0.05	−1.67

^10^ conducted a comprehensive study of the strain rate effect on the mechanical properties of cervical spine ligaments collected from a younger donor population. Their study revealed a significant stiffness dependency on the strain rate of all ligaments except the LF. In contrast, in our study, the LF exhibited the greatest increase in stiffness with the strain rate increase (78%), which was essentially greater than in^10^ (22%) and lower than in^12^ (265%). Our result corresponds well to^1^ under low-strain rate and to^9^ under high-strain rate, see Fig. 11. The stiffness increase observed for the strain rate of 50 s⁻¹ for the PLL and CL (Table 3) was slightly greater than the increase reported for the medium strain rate (20 s⁻¹) in^10^ (37% vs. 34% for the PLL and 48% vs. 43% for the CL)^10^. observed about two times greater strain rate effect for the high-strain rate (150–250 s⁻¹) than for the medium-strain rate. Generally, in the current study, a significant stiffness increase with the strain rate growth was obtained for all ligaments except ALL (Table 3), which exhibited approximately 19% lower stiffness at high-strain rate compared to quasi-static loading (Table 3). A 4% decrease in Young’s modulus, compared to a 19% decrease in stiffness, suggests that this decrease can be partially explained by the shorter length of the ligament samples used in the low-strain rate tests. Moreover, while in other studies^1,7,10^ the stiffness of the PLL was found to be greater than that of the ALL, in our low-strain rate tests the stiffness of the ALL was 25% greater than that of the PLL (Table 2). This suggests that relatively strong samples were used in the low-strain rate tests, which, combined with the small sample size and high biodiversity of the samples, may have resulted in the observed decrease in stiffness at higher strain rate in the ALL group. Furthermore, whereas in the other ligament groups the force at the second transition point was comparable for both strain rates, in the ALL group a 39% lower force was obtained in the high-strain rate tests compared to the low-strain rate tests (Table 2). The visco-hyperelastic constitutive model (10), which showed very good agreement with the experimental data (Table 7), predicts that the stiffness of the ligament increases during loading. Therefore, the significantly lower force at the second transition point may have reduced the stiffness of the ALL obtained in the high-strain rate tests due to the premature failure of some samples.

In the current study, a statistically insignificant increase in the failure force was observed only for the PLL and LF ligaments at high-strain rate tests (Table 3). Similarly, a smaller strain rate effect on the failure force than on stiffness was observed in^10^, where a significant increase was reported only for the CL. The greatest effect of strain rate on the failure force was obtained for the PLL (64%) and was greater than that reported for the medium strain rate (20 s⁻¹) in^10^ (46%). Thi result may be the effect of the relatively low failure load obtained for the PLL in the quasi-static tests compared to that obtained in^1^. In summary, comparison of our results with the rate dependence of mechanical properties documented by others^8–10,12^ indicates a smaller strain rate effect on the stiffness and failure load in the elderly population.

Direct comparison of the mechanical properties of spinal ligaments obtained at different strain rates is difficult. Therefore, in the current study, the visco-hyperelastic model taking into account the influence of collagen fiber reinforcement was proposed to describe rate-dependent, viscous mechanisms. In this formulation, the viscous component is adapted from approaches developed for collagenous tissues integrating a hyperelastic framework to capture the pronounced nonlinear response of ligament structures^30,35^. This approach contrasts with simplified linear-elastic models used in some HBMs, which do not account for fibrous tissues anisotropy or viscous effects^15^. Consistent with the strategy adopted in previous studies on soft tissues^35^. The material parameters are identified through a two-step curve-fitting process. First, hyperelastic parameters ($\:{C}_{10}$, $\:{C}_{2}$, and $\:{C}_{3}$) are extracted from quasi-static (0.5 s⁻¹) tests to represent the rate-independent behavior. Then, the viscous parameters ($\:{\eta\:}_{1}$, and $\:{\eta\:}_{2}$) are derived under high-strain-rate conditions (50 s⁻¹), capturing additional time-dependent effects. This division ensures that both the elastic and the viscous contributions are separated and accurately quantified.

The high coefficients of determination (Adj. *R*²) and the low error metrics (NRMSE, NMAE) reported in Table 4-Table 7 and results illustrated in Fig. 7-Fig. 10 confirm that the present visco-hyperelastic model accurately characterizes the nonlinear and time-dependent behavior of the tested ligaments. The worst goodness-of-fit metrics were obtained for the mean-parameter hyperelastic model for the CL and ALL (Table 5), which may overestimate the static ligament response at stretches greater than 1.2 and 1.25, respectively (see Fig. 8). However, this had no effect on the goodness-of-fit for the visco-hyperelastic mean-parameter model, since the stretch for the second transition point was approximately 1.2 in the dynamic tests. It is worth noting that the second transition point usually serves as the onset of the collagen fiber failure process^1^. The elevated goodness-of-fit metrics indicate that the model captures both the initial low-strain response (largely governed by the matrix) and the subsequent, more pronounced fiber-dominated regime.

Although the present results extend the knowledge of cervical spinal ligament behavior at both quasi-static and dynamic strain rates, certain limitations should be noted. First, all tests were carried out under uniaxial tension, reflecting the dominant loading mode of these ligaments but not necessarily replicating the full complexity of in vivo conditions. In practice, conducting multi-axial or off-axis tests for such small spinal ligaments is exceedingly difficult or infeasible, primarily due to geometric and mechanical constraints. Second, the study’s sample size could be expanded to improve statistical power and capture inter-individual variability more comprehensively. Third, the donor cohort ranged from 70 to 90 years of age, so extrapolating these results to younger populations or pathological cases should be done with caution. Finally, future work might involve exploring loading rates faster than 50 s⁻¹ to further clarify ligament behavior under severe dynamic conditions, such as those experienced during high-speed impacts. Furthermore, extending the visco-hyperelastic model to the sub-failure region would allow describing ligament behavior up to the failure point. Addressing these issues would help refine and validate the visco-hyperelastic model for broader applications in cervical spine biomechanics and injury prevention.

## Conclusions

This study investigated the mechanical behavior of four human cervical spine ligaments (ALL, PLL, LF, and CL) from older adult donors (70–90 years) under both low-strain (0.5 s⁻¹) and high-strain (50 s⁻¹) rate loading.

Key conclusions can be summarized as follows:

- **Strain-rate sensitivity**: Ligaments such as the PLL, LF, and CL showed statistically significant increases in stiffness and elastic modulus under rapid loading. Conversely, the ALL in this study presented slightly lower stiffness at 50 s⁻¹ than at 0.5 s⁻¹. An increase in the failure force was observed only for the PLL and LF at 50 s⁻¹.
- **Age-related variations**: Meta-regression indicated negative coefficients for stiffness and failure force with increasing donor age in certain cases. This finding reinforces the need to incorporate age-specific data into both clinical practice and computational models, particularly for geriatric populations.
- **Constitutive modeling**: The two-step fitting protocol, extracting hyperelastic parameters from quasi-static experiments and viscous parameters from high-strain rate tests, was shown to be effective, with mean-parameter models accurately describing the average ligament response up to the second transition point.
- **Applications and future work**: The presented stress–stretch data and material parameters can enhance FE and HBMs, potentially improving injury prediction in scenarios such as automotive crashes. Future research should focus on larger sample sizes, a broader range of donor demographics, and higher loading rates (> 50 s⁻¹) to further refine constitutive models and better capture ligament behavior under extreme conditions.

## Appendix

Table 9 and 10

**Table 9:** Geometry of ligaments expressed as medians and quartiles (Q1 - 25%, Q3- 75%)

**Lig.**	*Strain Rate*	*Area*, mm^2^			*Length, *mm			*Width, *mm		
		*Median*	*Q1*	*Q3*	*Median*	*Q1*	*Q3*	*Median*	*Q1*	*Q3*
**ALL**	Low	13.4	11.2	14.4	7.2	5.1	7.6	14.7	13.4	15.7
**ALL**	High	14	13.5	14.6	7.9	7.7	8.7	20.1	19.2	21.2
**PLL**	Low	14.2	12.5	15	6	5.3	7.4	14.5	13.7	15.1
**PLL**	High	15.4	12.7	16	9.5	7.2	10.2	17.7	17.7	19.1
**LF**	Low	49.4	49.1	54	9.7	9	10.8	13.6	11.4	13.8
**LF**	High	48.3	46.8	51	8.9	8.6	11	18.2	18	20
**CL**	Low	46	41.6	50.4	6.9	6.7	7.5	29	27.9	32.4
**CL**	High	48.6	42.7	53.7	7.3	7	8.2	23.6	22.1	24.3

**Table 10:** Mechanical properties of ligaments expressed as medians and quartiles

Lig.	*Strain Rate*	*Stiffness,* N/mm			*E,* MPa			F_f_, N			$P_{(11,f)},$ MPa		
		*Median*	*Q1*	*Q3*	*Median*	*Q1*	*Q3*	*Median*	*Q1*	*Q3*	*Median*	*Q1*	*Q3*
**ALL**	Low	101.4	91.3	116.2	54.2	46.1	61.8	201.8	153.9	250.2	14.9	13.8	19
**ALL**	High	80	64.2	106.3	43.3	40.2	53.6	146.2	134	207.5	14.2	9.3	15.2
**PLL**	Low	82.3	67.6	104.3	40.3	32.4	41.8	140.4	119.5	173.4	11.2	7.4	14.4
**PLL**	High	126	94.8	140.9	68.4	60.3	93.1	216.5	157.3	264.3	13.5	11.4	20.9
**LF**	Low	52	48.6	71.3	9.5	8.9	15.4	108.2	85.1	167.9	2.1	1.8	3.2
**LF**	High	113	83.2	132	20.8	15.3	26.8	151.7	123.3	179.4	3.2	2.8	4.2
**CL**	Low	55.1	38.1	70.7	8.4	6.3	11.6	113.1	93.5	162.6	2.8	2.1	3.6
**CL**	High	70.3	59.1	93.6	11.5	8.8	15.3	111.2	79.7	139.2	2.4	1.6	3

## Data Availability

The datasets used and/or analyzed during the current study are available from the corresponding author upon reasonable request. Raw data contain identifiable donor information and are therefore not publicly deposited to protect participant confidentiality.
